# Supplementary material for: Cavity electromechanics with parametric mechanical driving
Source: Nat Commun. 2020 Mar 27;11:1589. doi: 10.1038/s41467-020-15389-4 (PMC7101360; doi:10.1038/s41467-020-15389-4)
Supplement: Supplementary file 1 — Supplementary Information [file 41467_2020_15389_MOESM1_ESM.pdf]

Supplementary Material for:

Cavity electromechanics with parametric mechanical driving

Bothner *et al.*

SUPPLEMENTARY FIGURE 1: DEVICE FABRICATION

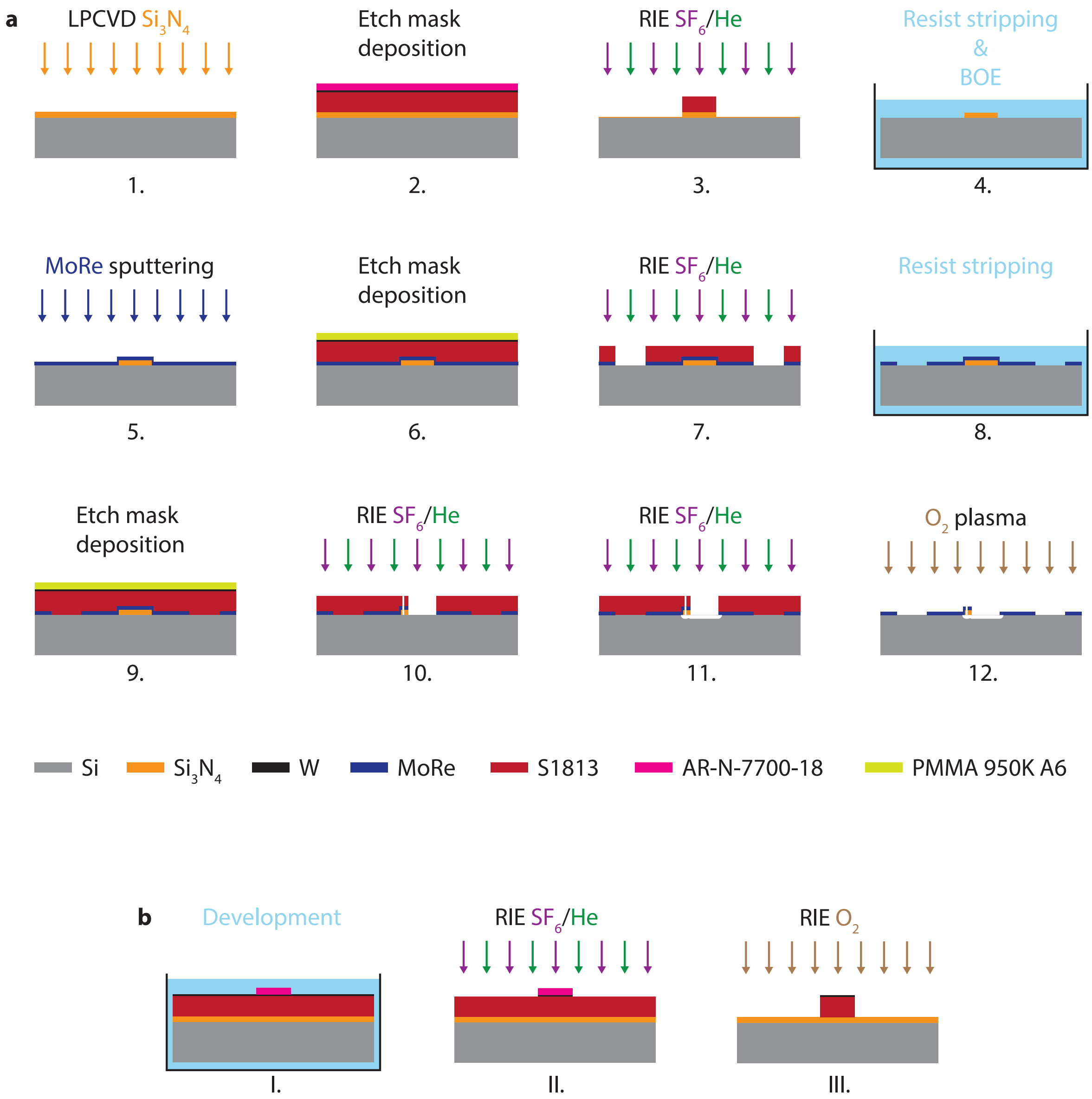

Supplementary Figure 1. **Schematic device fabrication.** **a** Fabrication steps 1. to 4. are the deposition and patterning of the Si_3N_4 patches, in steps 5. to 8. the deposition and patterning of the superconducting microwave structures is completed and the nanobeam patterning and release is done as steps 9. to 12. **b** Sub-steps between steps 2. and 3. of **a**. Equivalent steps are performed between 6. and 7. and between 9. and 10. of **a**. Dimensions are not to scale. A description of the individual steps is given in the methods section of the main text.

SUPPLEMENTARY NOTE 1: MEASUREMENT SETUP

Supplementary Fig. 2 shows a schematic of the measurement setup configurations, which we used for the experiments reported in this paper. All experiments were carried out in a dilution refrigerator with base temperature $T_\mathrm{b} = 15\,\mathrm{mK}$, cf. Supplementary Fig. 2**a**. The sample was mounted into a radiation tight copper housing and connected to two coaxial high-frequency lines. By means of a bias-tee, the center conductor of the coaxial line was also connected to

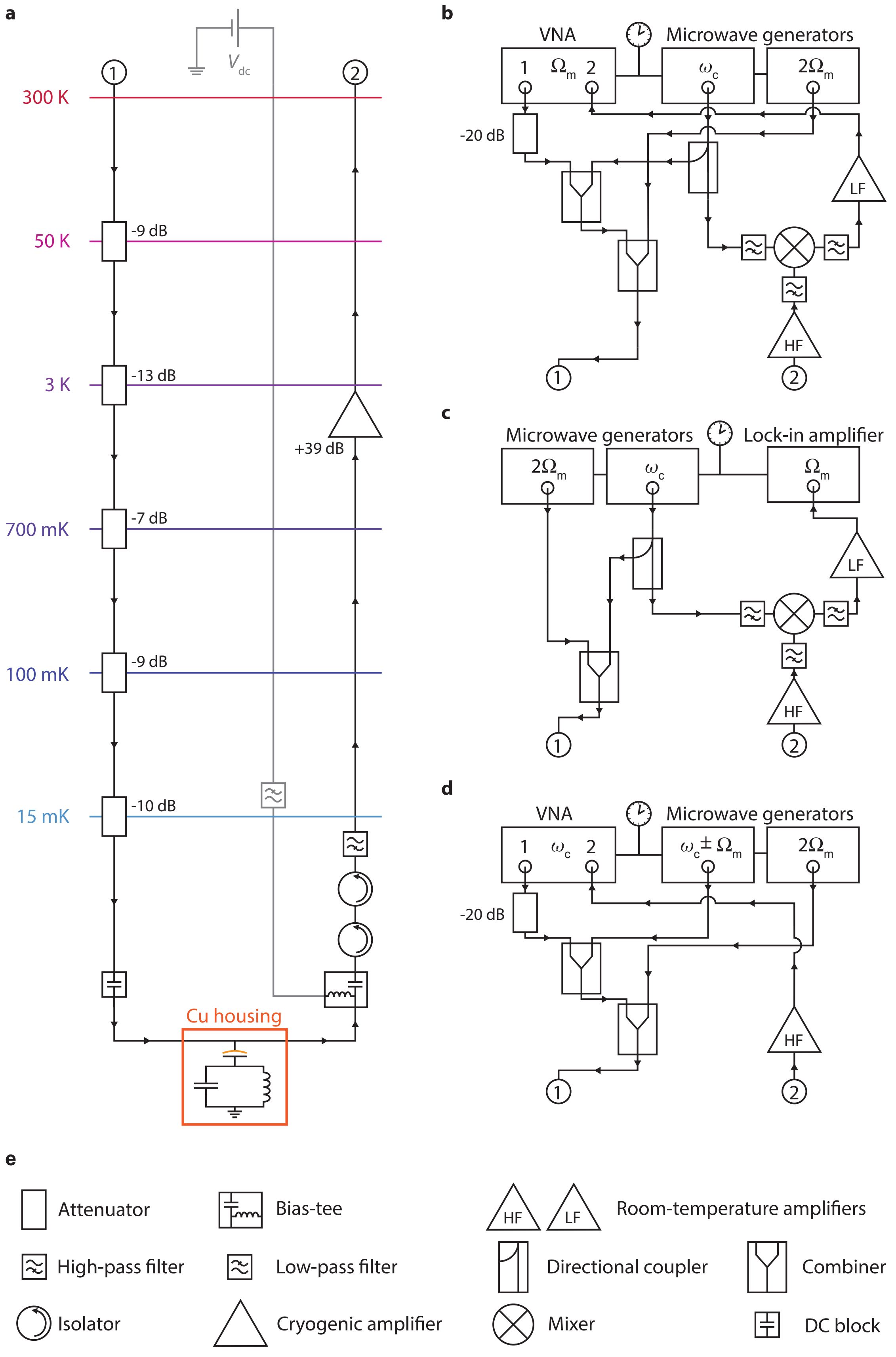

Supplementary Figure 2. **Schematic of the measurement setup.** Details are given in Supplementary Note 2.

a DC wire and a voltage source, which allowed for DC access to the sample. The input line was heavily attenuated to equilibrate the thermal radiation on the line to the refrigerator base temperature. In order to isolate the sample from the noise of the cryogenic amplifier on the output line, we used two isolators in series on the milliKelvin plate.

Outside of the refrigerator, we used different configurations of microwave signal sources and high-frequency electronics for the three experiments presented here. All three are shown in Supplementary Figs. 2**b**,**c** ,and **d**, where the setup for the mechanical parametric amplification experiment is shown in **b**, the setup for the thermomechanical noise squeezing is shown in **c** and the configuration for the parametric microwave amplification in **d**. For all experiments, the microwave sources and vector network analyzers (VNA) as well as the lock-in amplifier used a single reference clock of one of the devices.

Supplementary Fig. 2**e** provides a symbol legend for **a** to **d**.

SUPPLEMENTARY NOTE 2: CAVITY CHARACTERIZATION

The cavity model

The cavity used in this experiment is a quarter-wavelength ($\lambda/4$) transmission line cavity, capacitively side-coupled to a microwave feedline via a coupling capacitor C_c at the open end and shorted to ground at the other end. Cavity and feedline have the characteristic impedance Z_0 and the cavity has length l and resonance frequency ω_c. The cavity can be modeled around its fundamental mode resonance by a lumped element RLC circuit with the equivalent capacitor, inductor and resistor

$$C = \frac{C'l}{2}, \qquad L = \frac{8}{\pi^2}L'l, \qquad R = Z_0\alpha l \tag{1}$$

respectively. Here, C' and L' denote capacitance and inductance of the transmission line per unit length and α is the line attenuation constant.

For a capacitively coupled parallel RLC circuit, the ideal S_{21} response function is in the high-Q approximation given by

$$S_{21} = 1 - \frac{\kappa_\mathrm{e}}{\kappa_\mathrm{i} + \kappa_\mathrm{e} + 2i\Delta} \tag{2}$$

with the internal and external decay rates

$$\kappa_\mathrm{i} = \frac{1}{R(C + C_\mathrm{c})}, \qquad \kappa_\mathrm{e} = \frac{\omega_\mathrm{c}^2 C_\mathrm{c}^2 Z_0}{2(C + C_\mathrm{c})} \tag{3}$$

and the detuning from the resonance frequency

$$\Delta = \omega - \omega_\mathrm{c}, \qquad \omega_\mathrm{c} = \frac{1}{\sqrt{L(C + C_\mathrm{c})}}. \tag{4}$$

Cavity parameter extraction

Microwave cables and microwave elements such as attenuators, circulators and amplifiers cause the measured resonance line to deviate from Eq. (2) due to frequency dependent attenuation and cable interferences. To model the measured complex scattering parameter S_{21}, we use the modified response

$$S_{21} = (\alpha_0 + \alpha_1\omega)\left(1 - \frac{\kappa_\mathrm{e} e^{i\theta}}{\kappa_\mathrm{i} + \kappa_\mathrm{e} + 2i\Delta}\right) e^{i(\beta_1\omega + \beta_0)} \tag{5}$$

where we consider a modification of the background signal and phase by using the frequency dependent complex scaling factor

$$(\alpha_0 + \alpha_1\omega) \cdot e^{i(\beta_1\omega + \beta_0)} \tag{6}$$

and also include an additional rotation of the complex resonance circle around its anchor point by the phase factor $e^{i\theta}$.

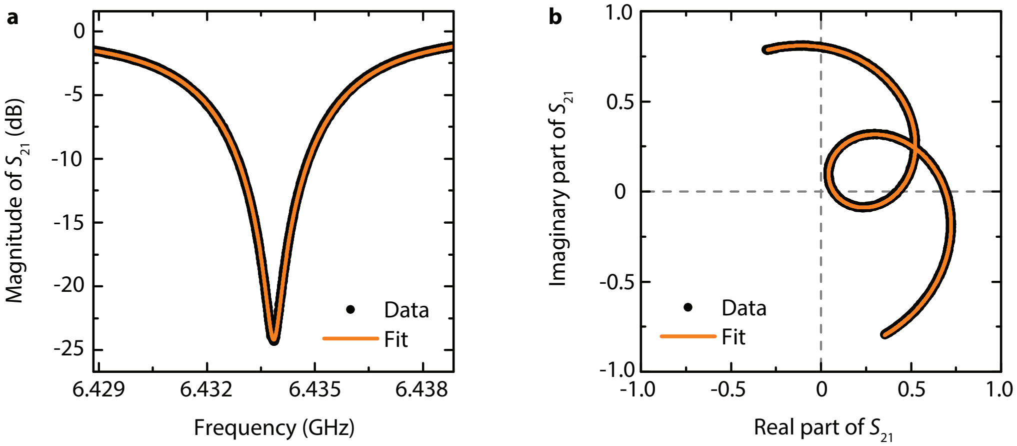

Supplementary Figure 3. **Fitting the resonance line and extraction of the relevant parameters.** **a** shows the magnitude of S_{21} and **b** the response in the complex plane. In both panels, data are shown as black circles and the fit as orange line.

Supplementary Fig. 3 shows an experimentally determined resonance curve in both, magnitude (**a**) and the complex plane (**b**), in direct comparison with the fit which we obtained using Eq. (5). Both curves are normalized by $\alpha_0+\alpha_1\omega_c$, i.e., by the background value at the fitted resonance frequency. From the fit, we extract $\kappa_i = 2\pi \cdot 370\,\mathrm{kHz}$, $\kappa_e = 2\pi \cdot 5.7\,\mathrm{MHz}$, and $\omega_c = 2\pi \cdot 6.4339\,\mathrm{GHz}$. Therefore, the cavity is highly overcoupled with a coupling efficiency $\eta = \kappa_e/(\kappa_i+\kappa_e) = 0.94$.

Cavity parameters vs sideband drive power

In the parts of the experiments, where we investigate optomechanically induced transparency and demonstrate microwave amplification, we add a high-power microwave tone on one of the cavity sidebands. This strong tone slightly modifies the cavity linewidths and the cavity resonance frequency. In Supplementary Fig. 4, we plot the resonance frequency (**a**) as well as external (**b**) and internal (**c**) cavity linewidths vs intracavity photon number as extracted from fitting the corresponding curves with Eq. (5). The frequency of the sideband drive was set to $\omega_d \approx \omega_c \pm \Omega_m$ and kept fixed for all powers. The deviation of the cavity resonance frequency from the low-power value, however, is given by maximally $0.007(\kappa_i+\kappa_e)$, i.e., less than one percent of the linewidth, and therefore we consider it as negligible and keep a fixed frequency for all sideband drives in our experiments.

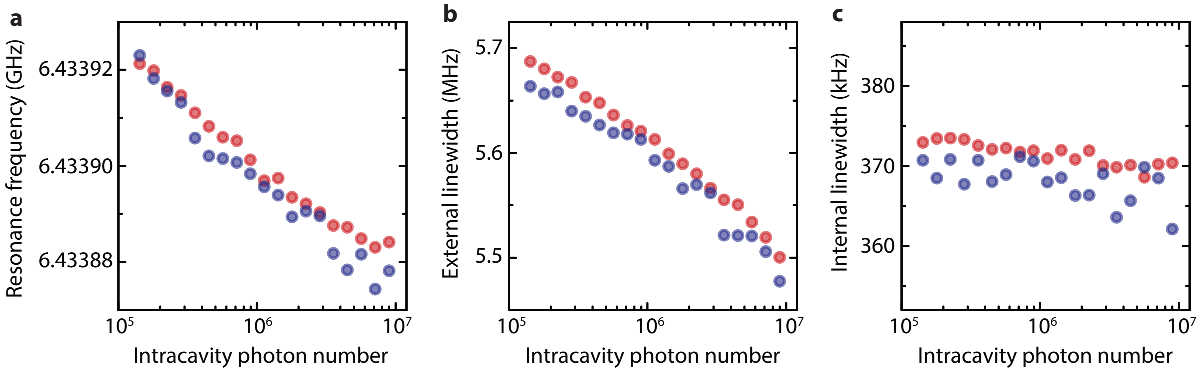

Supplementary Figure 4. **Cavity parameters in presence of a sideband drive vs intracavity photon number.** Red data points correspond to a drive at $\omega \sim \omega_c - \Omega_m$, blue data points to a drive at $\omega \sim \omega_c + \Omega_m$.

The intracavity photon number is calculated using

$$n = \frac{2P_{\mathrm{in}}}{\hbar\omega_d}\frac{\kappa_e}{\kappa^2+4\Omega_m^2}, \tag{7}$$

where $P_{\rm in}$ is the input power on the chip feedline, $\omega_{\rm d}$ is the drive frequency and we assume a detuning of the drive tone from the cavity resonance by one mechanical frequency $\Delta = \pm\Omega_{\rm m}$.

Coupling capacitance and characteristic impedance

With the formula for the external decay rate for a capacitively side-coupled RLC circuit

$$\kappa_{\rm e} = \frac{\pi\omega_{\rm c}C_{\rm c}^2}{8\sqrt{C(C+C_{\rm c})^3}} \tag{8}$$

the calculated capacitance per unit length $C' = 165\,$pF/m for our coplanar waveguide geometry (center conductor width $S = 10\,\mu$m, gap width $W = 6\,\mu$m, substrate permittivity $\epsilon_{\rm r} = 11.6$) and the cavity length $l = 3450\,\mu$m, we can determine the equivalent capacitance $C = 285\,$fF and the coupling capacitance $C_{\rm c} \approx 14\,$fF. In the expression for $\kappa_{\rm e}$, we have used that the resonance impedance of the equivalent RLC circuit can be expressed by $Z_{\rm r} = \sqrt{L/C} = \frac{4}{\pi}Z_0$.

With the value for the capacitances, we calculate the equivalent inductance $L = 2\,$nH, the inductance per unit length $L' = 731\,$nH/m, which is considerably larger than the calculated geometric inductance per unit length $L'_{\rm g} = 424\,$nH/m due to kinetic contributions, and finally the characteristic impedance of feedline and cavity as $Z_0 = 66.6\,\Omega$.

SUPPLEMENTARY NOTE 3: THEORY OF OPTOMECHANICAL MOTION DETECTION

When a microwave signal is sent to the cavity on resonance, the ideal response is given by

$$V(t) = V_\omega\left(\frac{\kappa_{\rm i}}{\kappa_{\rm i}+\kappa_{\rm e}}\right)e^{i\omega t}. \tag{9}$$

If the resonance frequency is modulated by mechanical motion, i.e., $\omega_{\rm c} = \omega_{\rm c} - G_0x(t)$ with the cavity pull $G_0 = -\frac{\partial\omega_{\rm c}}{\partial x}$, and assuming that $x(t)$ is a real-valued function and transients are small ($\kappa/\Omega_{\rm m} \approx 4$), we get

$$V(t) = V_\omega\left(\frac{\kappa_{\rm i}+2iG_0x(t)}{\kappa_{\rm i}+\kappa_{\rm e}+2iG_0x(t)}\right)e^{i\omega t} \tag{10}$$

$$\approx V_\omega\left(\frac{\kappa_{\rm i}}{\kappa_{\rm i}+\kappa_{\rm e}} + 2iG_0\frac{\kappa_{\rm e}}{(\kappa_{\rm i}+\kappa_{\rm e})^2}x(t)\right)e^{i\omega t}. \tag{11}$$

where the approximation in the last step was done for $G_0^2x^2 \ll (\kappa_{\rm i}+\kappa_{\rm e})^2$, i.e., the motion induced frequency shift is much smaller than the cavity linewidth.

With the Ansatz

$$x(t) = x_0\cos\Omega t = \frac{x_0}{2}\left(e^{i\Omega t}+e^{-i\Omega t}\right) \tag{12}$$

we get for the voltage response

$$V(t) = V_\omega\frac{\kappa_{\rm i}}{\kappa}e^{i\omega t} + iV_\omega G_0x_0\frac{\kappa_{\rm e}}{\kappa^2}\left(e^{i(\omega+\Omega)t}+e^{i(\omega-\Omega)t}\right). \tag{13}$$

To calculate the effect of mixing this response with a signal oscillating with ω, as we do in the experiment, we take the real part first given by

$$V_{\rm r}(t) = V_\omega\frac{\kappa_{\rm i}}{\kappa}\cos\omega t - V_\omega G_0x_0\frac{\kappa_{\rm e}}{\kappa^2}\left[\sin{(\omega+\Omega)t}+\sin{(\omega-\Omega)t}\right] \tag{14}$$

and multiply this with a mixing local oscillator $\cos{(\omega t+\gamma)}$ containing an arbitrary phase offset γ. The result is given by

$$V_{\rm f}(t) = \frac{V_\omega}{2}\frac{\kappa_{\rm i}}{\kappa}\cos\gamma - \frac{V_\omega}{2}\frac{\kappa_{\rm e}}{\kappa^2}G_0x_0\cos\Omega t\sin\gamma + ... \tag{15}$$

where we omitted frequency components oscillating with 2ω or $2\omega\pm\Omega$. Thus, if the mixer phase γ is different from zero or π, this technique will generate a signal with the frequency of the mechanical motion and the amplitude of this signal is proportional to the mechanical displacement amplitude. This way we detected both the mechanical amplitude amplification as well as the thermal noise squeezing in this work.

SUPPLEMENENARY NOTE 4: NANOWIRE CHARACTERIZATION

Nanowire tuning with a DC voltage

We describe the nanowire as a point-like mechanical harmonic oscillator, cf. also Supplementary Notes 6 and 8. When a DC voltage is applied to the center conductor of the transmission feedline, cf. Supplementary Fig. 2**a** and main paper Fig. 1, a static force is exerted to the nanowire and the equation of motion is given by

$$m\ddot{x} + m\Gamma_\mathrm{m}\dot{x} + k_\mathrm{m}x = \frac{1}{2}V_\mathrm{dc}^2\frac{\partial C_\mathrm{nw}}{\partial x}, \tag{16}$$

were x is the nanowire position, k_m is the intrinsic spring constant, m is the effective mass and Γ_m is the intrinsic damping or mechanical linewidth. The force will lead to a new nanowire equilibrium position x_0 which is defined by

$$k_\mathrm{m}x_0 = \frac{1}{2}V_\mathrm{dc}^2\frac{\partial C_\mathrm{nw}}{\partial x}\bigg|_{x_0}. \tag{17}$$

A Taylor approximation of the electrostatic force around the new equilibrium position x_0 gives

$$F_\mathrm{el} = \frac{1}{2}V_\mathrm{dc}^2\left[\frac{\partial C_\mathrm{nw}}{\partial x}\bigg|_{x_0} + \frac{\partial^2 C_\mathrm{nw}}{\partial x^2}\bigg|_{x_0}(x-x_0)...\right]. \tag{18}$$

Absorbing the new equilibrium position in a redefinition of the position coordinate x allows to write the full equation of motion as

$$m\ddot{x} + m\Gamma_\mathrm{m}\dot{x} + [k_\mathrm{m} + k_\mathrm{dc}]\,x = F(t) \tag{19}$$

where

$$k_\mathrm{dc} = -\frac{1}{2}V_\mathrm{dc}^2\frac{\partial^2 C_\mathrm{nw}}{\partial x^2}\bigg|_{x_0} \tag{20}$$

is the electrostatic spring constant and $F(t)$ is a possible additional external driving force. In general, the equilibrium position and the second derivative of the capacitance will depend on the applied DC voltage themselves.

From the equation of motion it follows that the mechanical resonance frequency is given by

$$\Omega_\mathrm{m} = \sqrt{\frac{k_\mathrm{m} + k_\mathrm{dc}}{m}} \tag{21}$$

$$= \Omega_\mathrm{m0}\sqrt{1 + \frac{k_\mathrm{dc}}{k_\mathrm{m}}} \tag{22}$$

where $\Omega_\mathrm{m0} = \sqrt{k_\mathrm{m}/m}$ is the intrinsic mechanical resonance frequency. Note that the electrostatic spring constant is negative and that the resonance frequency is shifted to lower values.

To characterize the mechanical oscillator, we drive it with an additional near-resonant harmonic voltage as described in Supplementary Note 6 and measure the resonance peak for different DC voltages. With an estimate for the mass of the nanobeam, we can extract the effective spring constant from the zero voltage resonance frequency. With the dimensions of the beam, its full mass is calculated by using the densities $\rho_\mathrm{SiN} = 3.2\,\mathrm{g\ cm^{-3}}$ and $\rho_\mathrm{MoRe} = 14.5\,\mathrm{g\ cm^{-3}}$ to be $m = 17\,\mathrm{pg}$.

From the resonance frequency $\Omega_\mathrm{m0} = 2\pi\cdot 1.478\,\mathrm{MHz}$, we can therefore extract the effective intrinsic spring constant $k_\mathrm{m} = 1.46\,\mathrm{N\ m^{-1}}$. In addition, we can calculate the electrical spring constant k_dc from here. The measured resonance frequency vs DC voltage in the negative voltage range is plotted in Supplementary Fig. 5**a** and a fit with Eq. (22) assuming $k_\mathrm{dc} \propto V_\mathrm{dc}^2$ describes the behaviour very accurately in the shown voltage range.

With Eq. (22) we can also calculate the electrostatic spring constant

$$k_\mathrm{dc} = k_\mathrm{m}\left(1 - \frac{\Omega_\mathrm{m}^2}{\Omega_\mathrm{m0}^2}\right) \tag{23}$$

from our data and $k_\mathrm{m} = 1\,\mathrm{N\ m^{-1}}$. The result is plotted in Supplementary Fig. 5**b** together with a line obtained from the fit in **a**.

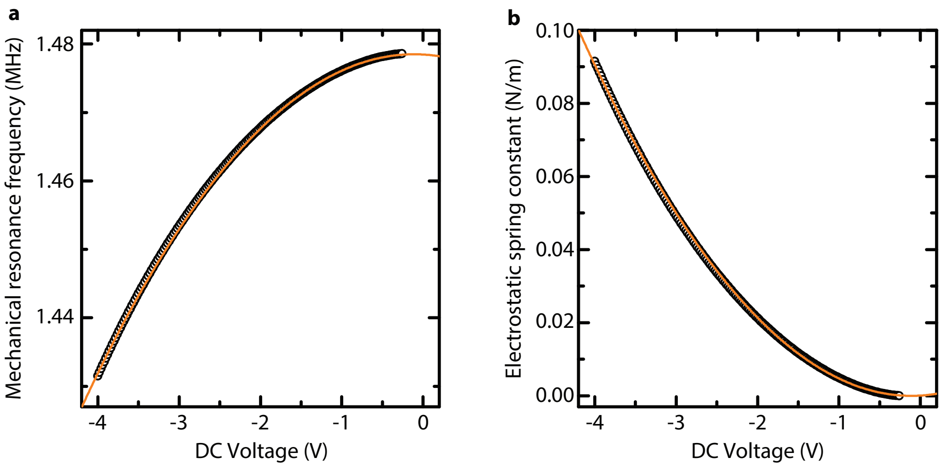

Supplementary Figure 5. **Mechanical resonance frequency tuning and electrostatic spring constant.** In **a** the measured resonance frequency is plotted vs applied DC voltage on the feedline (black circles). The orange line is a fit using Eq. (22) with $k_{\mathrm{dc}} \propto V_{\mathrm{dc}}^2$. In **b** the magnitude of the electrostatic spring constant calculated from the fit in **a** is shown as orange line. The experimental data (black circles) are calculated using Eq. (23). At the operation point of this paper $V_{\mathrm{dc}} = -4\,\mathrm{V}$, we obtain $k_{\mathrm{dc}} \approx -0.09\,\mathrm{N/m}$.

SUPPLEMENTARY NOTE 5: OPTOMECHANICAL DEVICE CHARACTERIZATION

Optomechanical coupling rate g_0

To calculate the optomechanical single-photon coupling rate

$$g_0 = -\frac{\partial \omega_\mathrm{c}}{\partial x} x_\mathrm{zpf} \tag{24}$$

we need the mechanical zero-point fluctuations, which we get from the resonance frequency and the effective nanowire mass as

$$x_\mathrm{zpf} = \sqrt{\frac{\hbar}{2m\Omega_\mathrm{m}}} = 18\,\mathrm{fm}. \tag{25}$$

To get the cavity pull parameter, we estimate from simulations and calculations the mechanical capacitance to be approximately $C_\mathrm{nw} = 2\,\mathrm{fF}$ and calculate

$$\frac{\partial \omega_\mathrm{c}}{\partial C_\mathrm{nw}} = -\frac{\omega_\mathrm{c}^3}{2} L = -2\pi \cdot 9.5 \cdot 10^{21}\,\mathrm{Hz}\ \mathrm{F}^{-1}. \tag{26}$$

The final quantity we need is

$$\frac{\partial C_\mathrm{nw}}{\partial x} \approx 5 \cdot 10^{-9}\,\mathrm{F}\ \mathrm{m}^{-1}, \tag{27}$$

which gives a cavity pull of

$$G_0 = -\frac{\partial \omega_\mathrm{c}}{\partial x} = 2\pi \cdot 48\,\mathrm{kHz}\ \mathrm{nm}^{-1}. \tag{28}$$

Putting everything together we get

$$g_0 = 2\pi \cdot 0.9\,\mathrm{Hz}. \tag{29}$$

Optomechanical coupling ratio

Resonance frequency and external coupling rate of our cavity are given by

$$\omega_\mathrm{c} = \frac{1}{\sqrt{L(C+C_\mathrm{c})}} \tag{30}$$

$$\kappa_\mathrm{e} = \frac{Z_0 C_\mathrm{c}^2}{2L(C+C_\mathrm{c})^2} \tag{31}$$

with the characteristic feedline impedance Z_0. Assuming that the cavity is highly overcoupled $\kappa_\mathrm{e}+\kappa_\mathrm{i} \approx \kappa_\mathrm{e}$ as in our device, the ratio of dissipative optomechanical coupling rate g_κ to dispersive optomechanical coupling rate g_ω is given by

$$\frac{g_\kappa}{g_\omega} = 2Z_0\omega_\mathrm{c}\frac{CC_\mathrm{c}}{C+C_\mathrm{c}} \approx 0.07, \tag{32}$$

which is small enough to neglect the dissipative optomechanical coupling contribution to first order throughout the paper. Therefore, we will restrict our theoretical calculations and device modeling to purely dispersive coupling.

Theory of optomechanically induced transparency and absorption without the resolved sideband limit

We model the system without the parametric driving by means of the classical, coupled equations of motion for the mechanical displacement x and the intracavity field amplitude α

$$\ddot{x} = -\Omega_\mathrm{m}^2 x - \Gamma_\mathrm{m}\dot{x} + \frac{1}{m}\left(F_\mathrm{r}+F_\mathrm{e}\right) \tag{33}$$

$$\dot{\alpha} = \left[i(\Delta + G_0 x) - \frac{\kappa}{2}\right]\alpha + \sqrt{\frac{\kappa_\mathrm{e}}{2}}S_\mathrm{in} \tag{34}$$

where external forces to the mechanical oscillator are expressed by F_e in the first equation and the radiation pressure force due to the intracavity field is given by

$$F_\mathrm{r} = \hbar G_0|\alpha|^2. \tag{35}$$

Further parameters in the equations are the cavity pull parameter $G_0 = -\partial\omega_\mathrm{c}/\partial x$, the detuning between a cavity drive and the cavity resonance frequency $\Delta = \omega_\mathrm{d} - \omega_\mathrm{c}$ and the total cavity linewidth $\kappa = \kappa_\mathrm{i} + \kappa_\mathrm{e}$. In the second equation, the field amplitude α is normalized such that $|\alpha|^2$ corresponds to the photon number in the cavity and the input field S_in is normalized such that $|S_\mathrm{in}|^2$ corresponds to the photon number flux of the input field.

Under the assumption that it is sufficient to consider only small deviations from the steady state solutions $\bar{x}, \bar{\alpha}$ of the full equations, i.e., $x = \bar{x} + \delta x, \alpha = \bar{\alpha} + \delta\alpha$, these two equations can be linearized as

$$\delta\ddot{x} = -\Omega_\mathrm{m}^2\delta x - \Gamma_\mathrm{m}\delta\dot{x} + \frac{\hbar G_0\bar{\alpha}}{m}\left(\delta\alpha + \delta\alpha^*\right) \tag{36}$$

$$\delta\dot{\alpha} = \left[i\bar{\Delta} - \frac{\kappa}{2}\right]\delta\alpha + iG_0\bar{\alpha}\delta x + \sqrt{\frac{\kappa_\mathrm{e}}{2}}S_\mathrm{p}. \tag{37}$$

where we omitted a possible external driving force F_e. Here, $\bar{\Delta} = \omega_\mathrm{d} - \omega_\mathrm{c} + G_0\bar{x}$ is the detuning from the modified resonance frequency, when the mechanical oscillator is pushed by radiation pressure to its new equilibrium position $\bar{x}$, and $\sqrt{\kappa_\mathrm{e}/2}S_\mathrm{p}$ with $S_\mathrm{p} = S_0 e^{-i\Omega t}$ $(\Omega = \omega - \omega_\mathrm{d})$ accounts for small additional drive fields or field fluctuations.

We solve these equations with the Ansatz

$$\delta\alpha = a_- e^{-i\Omega t} + a_+ e^{+i\Omega t} \tag{38}$$

$$\delta\alpha^* = a_-^* e^{+i\Omega t} + a_+^* e^{-i\Omega t} \tag{39}$$

$$\delta x = x_1 e^{-i\Omega t} + x_1^* e^{+i\Omega t} \tag{40}$$

and get as solution in high-Q_m approximation the modified mechanical response function

$$\chi_\mathrm{m}^\mathrm{eff} = \frac{1}{2m\Omega_\mathrm{m}}\frac{1}{\Omega_\mathrm{m} - \Omega - i\frac{\Gamma_\mathrm{m}}{2} + \Sigma'(\Omega_\mathrm{m})} \tag{41}$$

where

$$\Sigma'(\Omega_\mathrm{m}) = -ig^2\left[\chi_\mathrm{c}(\Omega_\mathrm{m}) - \chi_\mathrm{c}^*(-\Omega_\mathrm{m})\right]. \tag{42}$$

Here,

$$\chi_\mathrm{c} = \frac{1}{\frac{\kappa}{2} - i(\bar{\Delta} + \Omega)} \tag{43}$$

with $\bar{\Delta} = \omega_\mathrm{d} - \omega_\mathrm{c} + G_0\bar{x}$ represents the (modified) cavity response lineshape. From here on we use just Δ for $\bar{\Delta}$, as the difference is negligibly small in our experiment.

Expression (42) can be split into an imaginary and a real part $\Sigma' = \delta\Omega_\mathrm{m} - i\Gamma_\mathrm{o}/2$, of which the real part corresponds to a modification of the mechanical resonance frequency (optical spring)

$$\delta\Omega_\mathrm{m} = g^2\left[\frac{\Delta + \Omega_\mathrm{m}}{\frac{\kappa^2}{4} + (\Delta + \Omega_\mathrm{m})^2} + \frac{\Delta - \Omega_\mathrm{m}}{\frac{\kappa^2}{4} + (\Delta - \Omega_\mathrm{m})^2}\right] \tag{44}$$

and the imaginary part

$$\Gamma_\mathrm{o} = g^2\kappa\left[\frac{1}{\frac{\kappa^2}{4} + (\Delta + \Omega_\mathrm{m})^2} - \frac{1}{\frac{\kappa^2}{4} + (\Delta - \Omega_\mathrm{m})^2}\right] \tag{45}$$

represents an additional damping term (optical damping).

For the cavity amplitude, we find the solution

$$a_- = \chi_\mathrm{c}\left[1 + 2im\Omega_\mathrm{m}g^2\chi_\mathrm{c}\chi_\mathrm{m}^\mathrm{eff}\right]\sqrt{\frac{\kappa_\mathrm{e}}{2}}S_0 \tag{46}$$

which with $S_{21} = 1 - \sqrt{\frac{\kappa_\mathrm{e}}{2}}\frac{a_-}{S_0}$ can be directly translated into the full cavity response function in presence of a harmonic drive

$$S_{21} = 1 - \frac{\kappa_\mathrm{e}}{2}\chi_\mathrm{c}\left[1 + 2im\Omega_\mathrm{m}g^2\chi_\mathrm{c}\chi_\mathrm{m}^\mathrm{eff}\right]. \tag{47}$$

Drive on the red sideband

When the constant frequency drive is set to the red cavity sideband, i.e., $\Delta = -\Omega_\mathrm{m}$, and the probe tone is swept only very close to the cavity resonance, i.e., $\Omega = \Omega_\mathrm{m} + \Delta_\mathrm{m}$ with $\Delta_\mathrm{m} \ll \kappa$, the effective cavity susceptibility is given by

$$\chi_\mathrm{c} = \frac{2}{\kappa} \tag{48}$$

and the effective mechanical susceptibility can be approximated as

$$\chi_\mathrm{m}^\mathrm{eff} = -\frac{1}{m\Omega_\mathrm{m}}\frac{1}{2\Delta_\mathrm{m} + i\Gamma_\mathrm{eff}} \tag{49}$$

with

$$\Gamma_\mathrm{eff} = \Gamma_\mathrm{m} + \Gamma_\mathrm{o} = \Gamma_\mathrm{m}\left(1 + \mathcal{C}\frac{16\frac{\Omega_\mathrm{m}^2}{\kappa^2}}{1 + 16\frac{\Omega_\mathrm{m}^2}{\kappa^2}}\right) \tag{50}$$

where $\mathcal{C} = 4g^2/\kappa\Gamma_\mathrm{m}$ is the cooperativity.

The scattering parameter is given by

$$S_{21} = 1 - \frac{\kappa_\mathrm{e}}{\kappa}\left[1 - 4i\frac{g^2}{\kappa}\frac{1}{2\Delta_\mathrm{m} + i\Gamma_\mathrm{eff}}\right] \tag{51}$$

$$= \frac{\kappa_\mathrm{i}}{\kappa} + i\frac{\kappa_\mathrm{e}}{\kappa}\frac{\mathcal{C}\Gamma_\mathrm{m}}{2\Delta_\mathrm{m} + i\Gamma_\mathrm{eff}}. \tag{52}$$

and the transmitted power is described by a Lorentzian

$$|S_{21}|^2 = S_\mathrm{c} + \frac{\mathcal{C}\Gamma_\mathrm{m}}{4\Delta_\mathrm{m}^2 + \Gamma_\mathrm{eff}^2} S_\mathrm{om} \tag{53}$$

with the background value

$$S_\mathrm{c} = \frac{\kappa_\mathrm{i}^2}{\kappa^2} \tag{54}$$

and the optomechanical amplitude

$$S_\mathrm{om} = 2\frac{\kappa_\mathrm{i}\kappa_\mathrm{e}}{\kappa^2}\Gamma_\mathrm{eff} + \frac{\kappa_\mathrm{e}^2}{\kappa^2}\mathcal{C}\Gamma_\mathrm{m}. \tag{55}$$

Drive on the blue sideband

With $\Delta = +\Omega_\mathrm{m}$ and $\Omega \approx -\Omega_\mathrm{m} + \Delta_\mathrm{m}$ we get

$$\chi_\mathrm{m}^\mathrm{eff} = \frac{1}{m\Omega_\mathrm{m}} \frac{1}{2\Delta_\mathrm{m} + i\Gamma'_\mathrm{eff}} \tag{56}$$

where

$$\Gamma'_\mathrm{eff} = \Gamma_\mathrm{m} - \Gamma_\mathrm{o} = \Gamma_\mathrm{m}\left(1 - \mathcal{C}\frac{16\frac{\Omega_\mathrm{m}^2}{\kappa^2}}{1 + 16\frac{\Omega_\mathrm{m}^2}{\kappa^2}}\right). \tag{57}$$

As transmission parameter we thus get

$$S_{21} = 1 - \frac{\kappa_\mathrm{e}}{\kappa}\left[1 + 4i\frac{g^2}{\kappa}\frac{1}{2\Delta_\mathrm{m} + i\Gamma'_\mathrm{eff}}\right] \tag{58}$$

$$= \frac{\kappa_\mathrm{i}}{\kappa} - i\frac{\kappa_\mathrm{e}}{\kappa}\frac{\mathcal{C}\Gamma_\mathrm{m}}{2\Delta_\mathrm{m} + i\Gamma'_\mathrm{eff}} \tag{59}$$

and for the transmitted power

$$|S_{21}|^2 = S_\mathrm{c} + \frac{\mathcal{C}\Gamma_\mathrm{m}}{4\Delta_\mathrm{m}^2 + \Gamma'^2_\mathrm{eff}} S'_\mathrm{om} \tag{60}$$

with

$$S'_\mathrm{om} = -2\frac{\kappa_\mathrm{i}\kappa_\mathrm{e}}{\kappa^2}\Gamma_\mathrm{eff} + \frac{\kappa_\mathrm{e}^2}{\kappa^2}\mathcal{C}\Gamma_\mathrm{m} \tag{61}$$

OMIT, OMIA and cooperativity

In the experiment, we drive the cavity with a drive tone on one of the sidebands, i.e. at $\omega = \omega_\mathrm{c} \pm \Omega_\mathrm{m}$ and variable power. Then, we sweep a weak probe tone around the cavity resonance and measure the resulting optomechanically induced transparency or absorption. Supplementary Fig. 6 shows the resulting transparency and absorption windows for different drive powers, i.e., for different drive photon numbers inside the cavity. In **a**, the optomechanically induced transparency window for a drive on the red sideband $\omega = \omega_\mathrm{c} - \Omega_\mathrm{m}$ is shown and in **b** the corresponding data for a drive on the blue sideband $\omega = \omega_\mathrm{c} + \Omega_\mathrm{m}$. The different curves correspond to different drive powers (steps of 2 dB) or intracavity photon numbers, respectively.

From the amplitude of the Lorentzians, we can extract the cooperativity $\mathcal{C}$ and the total coupling rate g. The extracted values for the highest power data are given in Supplementary Fig. 6.

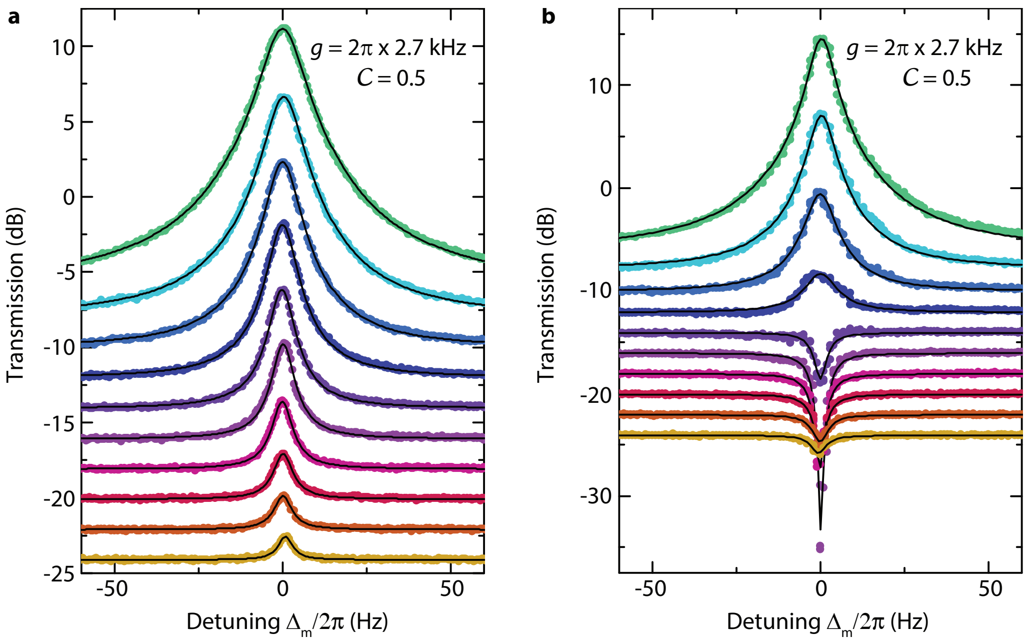

Supplementary Figure 6. **Optomechanically induced transparency and absorption.** For this experiment we drive the cavity with a strong drive tone on either the red sideband $\omega = \omega_\mathrm{c} - \Omega_\mathrm{m}$ (**a**) or on the blue sideband $\omega = \omega_\mathrm{c} + \Omega_\mathrm{m}$ (**b**). Then we sweep a weak probe signal around $\Delta_\mathrm{m} = \omega - \omega_\mathrm{c}$. In **a**, a Lorentzian shaped peak corresponding to exciting the mechanical resonator appears and grows with increasing sideband drive power in both, height and width. In **b**, the cavity response for a drive on the blue sideband is shown. For the lower powers, optomechanically induced absorption appears, i.e., a narrow absorption dip in the cavity minimum. For higher powers, this dip turns into a transparency peak as well. The lowest line in both (baseline at $-24\,\mathrm{dBm}$), **a** and **b**, corresponds to the lowest drive power and subsequent lines are manually upshifted by $2\,\mathrm{dB}$ each for better visibility. For both plots, the difference in drive power between subsequent lines is $2\,\mathrm{dB}$, where the largest power corresponds to an intracavity drive photon number of $\sim 9 \cdot 10^6$.

Effective mechanical linewidth

The effective linewidth of the mechanical oscillator in an optomechanical system is given by the sum of the intrinsic linewidth Γ_m and the optical linewidth Γ_o due to dynamical backaction. When extracting the linewidths from the data in Supplementary Fig. 6, we find that we can best describe the overall dependence on the intracavity photon number by modelling a photon number dependent intrinsic linewidth $\Gamma_\mathrm{m}(n_\mathrm{c})$, which might be caused by heating of the nanobeam, which is coupled directly to the feeedline. When we model the intrinsic linewidth as shown by the dashed gray line in Supplementary Fig. 7**a**, we find very good agreement between the theoretical lines of $\Gamma_\mathrm{eff} = \Gamma_\mathrm{m} + \Gamma_\mathrm{o}$ and the data points. We use the phenomenological functional description $\Gamma_\mathrm{m}(n_\mathrm{c}) = \Gamma_\mathrm{m0} + \gamma_1 \arctan \gamma_2 n_\mathrm{c}$ here with γ_i constant parameters adjusted to best describe the experimental data. Calculating the peak transmission for this linewidth dependence in combination with the photon number, g_0, κ_i and κ_e, gives excellent agreement between the theoretical curves and the data as shown in **b**.

SUPPLEMENTARY NOTE 6: THEORY OF PARAMETRIC MECHANICAL AMPLITUDE AMPLIFICATION

Similar to the description given in Ref. [1], we model the nanowire as mechanical harmonic oscillator with the effective equation of motion of a point-like particle having the position coordinate x

$$m\ddot{x} + m\Gamma_\mathrm{m}\dot{x} + k_\mathrm{m}x = F(t). \tag{62}$$

Here, m is the effective mass of the nanowire, Γ_m is its damping rate, k_m is the mechanical spring constant and $F(t)$ is a time dependent external driving force. When a time-dependent voltage $V(t)$ is applied to the center conductor of the microwave feedline, the nanowire experiences a corresponding electrical force

$$F_\mathrm{el} = \frac{1}{2}\frac{\partial C_\mathrm{nw}}{\partial x}V^2 \tag{63}$$

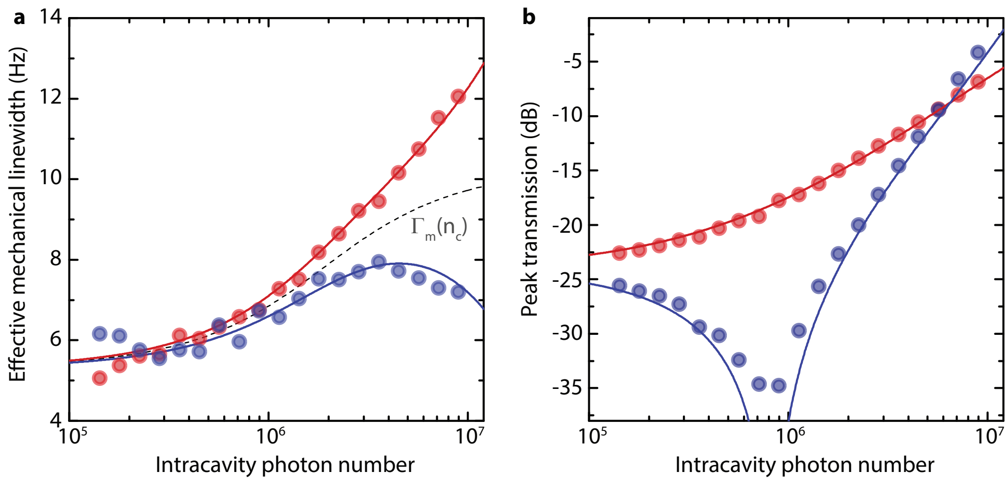

Supplementary Figure 7. **Effective mechanical linewidth and OMIT peak transmission vs intracavity photon number.** **a** shows the effective mechanical linewidth extracted from the data in Supplementary Fig. 6 as points, red points for the red sideband, blue points for the blue. The dashed line shows the model for a power-dependent intrinsic linewidth $\Gamma_\mathrm{m}(n_\mathrm{c})$ and the red and blue lines show the resulting calculated effective linewidth including dynamical backaction. In **b**, the peak transmission is plotted, circles correspond to experimental values and lines to theoretical calculations based on $\Gamma_\mathrm{m}(n_\mathrm{c})$, g_0, n_c, κ_i, κ_e and Eqs. (53) and (60).

where C_nw is the capacitance between the nanowire and the center conductor of the cavity (DC ground). In our experiment, the total voltage applied to the center conductor (without the microwave tone) is given by

$$V(t) = V_\mathrm{dc} + V_0 \cos(\Omega t + \phi_\mathrm{p}) + V_{2\Omega} \sin 2\Omega t \tag{64}$$

which corresponds to a total force of

$$F_\mathrm{el}(t) = \tfrac{1}{2}\tfrac{\partial C_\mathrm{nw}}{\partial x}\Big[V_\mathrm{dc}^2 + V_0^2 \cos^2(\Omega t + \phi_\mathrm{p}) + V_{2\Omega}^2 \sin^2 2\Omega t \tag{65}$$

$$+2V_\mathrm{dc}V_0 \cos(\Omega t + \phi_\mathrm{p}) + 2V_\mathrm{dc}V_{2\Omega} \sin 2\Omega t \tag{66}$$

$$+V_0 V_{2\Omega} \cos(\Omega t + \phi_\mathrm{p}) \sin 2\Omega t\Big] \tag{67}$$

Here, V_dc is a static voltage, V_0 is the voltage peak amplitude of a harmonic drive close to resonance with frequency Ω and $V_{2\Omega}$ is the corresponding amplitude of the parametric drive voltage with twice the frequency of the near-resonant drive. We consider a phase shift between the near-resonant and the parametric drive by the phase ϕ_p in the near-resonant term.

In the experiment, we have used $V_\mathrm{dc} = -4\,\mathrm{V}$, $V_{2\Omega} \leq 100\,\mu\mathrm{V}$ and $V_0 \approx 100\,\mathrm{nV}$. Keeping only the leading terms under these conditions, we get

$$F_\mathrm{el} \approx \frac{1}{2}\frac{\partial C_\mathrm{nw}}{\partial x}\left[V_\mathrm{dc}^2 + 2V_\mathrm{dc}V_0 \cos(\Omega t + \phi_\mathrm{p}) + 2V_\mathrm{dc}V_{2\Omega} \sin 2\Omega t\right]. \tag{68}$$

A Taylor approximation to first order in x around the equilibrium position x_0 gives

$$F_\mathrm{el} \approx \frac{1}{2}\left[V_\mathrm{dc}^2 + 2V_\mathrm{dc}V_0 \cos(\Omega t + \phi_\mathrm{p}) + 2V_\mathrm{dc}V_{2\Omega} \sin 2\Omega t\right]\left[\left.\frac{\partial C_\mathrm{nw}}{\partial x}\right|_{x_0} + \left.\frac{\partial^2 C_\mathrm{nw}}{\partial x^2}\right|_{x_0} \cdot (x - x_0) + ...\right] \tag{69}$$

The first order terms proportional to $x - x_0$ can now be regarded as an electrostatic spring force with the spring constant

$$k_\mathrm{el}(t) = \underbrace{-\frac{1}{2}\left.\frac{\partial^2 C_\mathrm{nw}}{\partial x^2}\right|_{x_0} V_\mathrm{dc}^2}_{=k_\mathrm{dc}} \underbrace{-\left.\frac{\partial^2 C_\mathrm{nw}}{\partial x^2}\right|_{x_0} V_\mathrm{dc}V_{2\Omega} \sin 2\Omega t}_{=k_\mathrm{p}} \tag{70}$$

where we have omitted the $\cos(\Omega t + \phi_\mathrm{p})$ term due to its smallness and the reduced effect of resonant parametric modulations compared to a 2Ω-term []. Similarly, we can omit the 2Ω-term in the remaining driving force and after absorbing the remaining static force into a redefinition of the equilibrium position $x_0 = 0$ we finally get

$$m\ddot{x} + \Gamma_\mathrm{m}\dot{x} + [k_0 + k_\mathrm{p}\sin 2\Omega t]\, x = F_0 \cos(\Omega t + \phi_\mathrm{p}) \tag{71}$$

with

$$k_0 = k_\mathrm{m} + k_\mathrm{dc}, \qquad F_0 = V_\mathrm{dc} V_0 \left.\frac{\partial C_\mathrm{nw}}{\partial x}\right|_{x_0}. \tag{72}$$

This is the well-known equation of motion for a parametrically modulated harmonic oscillator [1].

With the transformations

$$\Omega_1 = \Omega_\mathrm{m}\left[\left(1 - \frac{1}{4Q_\mathrm{m}^2}\right)^{1/2} + \frac{i}{2Q_\mathrm{m}}\right], \tag{73}$$

$$A = \dot{x} + i\Omega_1^* x \tag{74}$$

$$A^* = \dot{x} - i\Omega_1 x \tag{75}$$

we rewrite the equation of motion as

$$\dot{A} = i\Omega_1 A + i\frac{k_\mathrm{p}\sin 2\Omega t}{m}\frac{A - A^*}{\Omega_1^* + \Omega_1} + \frac{F_0}{m}\cos(\Omega t + \phi_\mathrm{p}). \tag{76}$$

With the Ansatz

$$A = A_0 e^{i\Omega t}, \tag{77}$$

and the high-Q_m approximations

$$\Omega_1^* + \Omega_1 \approx 2\Omega_\mathrm{m}, \tag{78}$$

$$\Omega_1 - \Omega \approx i\frac{\Omega_\mathrm{m}}{2Q_\mathrm{m}} - \Delta_\mathrm{m} \tag{79}$$

where $\Delta_\mathrm{m} = \Omega - \Omega_\mathrm{m}$, we find in rotating wave approximation

$$\frac{\Omega_\mathrm{m}}{2Q_\mathrm{m}}A_0 + i\Delta_\mathrm{m}A_0 + \frac{k_\mathrm{p}}{4m\Omega_\mathrm{m}}A_0^* - \frac{F_0}{2m}e^{i\phi_\mathrm{p}} = 0. \tag{80}$$

We can solve this equation for the real and the imaginary part of A_0. Setting $x(t) = x_1\cos\Omega t + x_2 \sin\Omega t$ and using $x_1 = \mathrm{Im}(A_0)/\Omega_\mathrm{m}$, $x_2 = \mathrm{Re}(A_0)/\Omega_\mathrm{m}$ we get

$$x_1 = F_0\frac{Q_\mathrm{m}}{k_0}\left[\frac{\left(1 + \frac{Q_\mathrm{m}k_\mathrm{p}}{2k_0}\right)\sin\phi_\mathrm{p} - \frac{2Q_\mathrm{m}\Delta_\mathrm{m}}{\Omega_\mathrm{m}}\cos\phi_\mathrm{p}}{1 - \frac{Q_\mathrm{m}^2 k_\mathrm{p}^2}{4k_0^2} + \frac{4Q_\mathrm{m}^2\Delta_\mathrm{m}^2}{\Omega_\mathrm{m}^2}}\right] \tag{81}$$

$$x_2 = F_0\frac{Q_\mathrm{m}}{k_0}\left[\frac{\left(1 - \frac{Q_\mathrm{m}k_\mathrm{p}}{2k_0}\right)\cos\phi_\mathrm{p} + \frac{2Q_\mathrm{m}\Delta_\mathrm{m}}{\Omega_\mathrm{m}}\sin\phi_\mathrm{p}}{1 - \frac{Q_\mathrm{m}^2 k_\mathrm{p}^2}{4k_0^2} + \frac{4Q_\mathrm{m}^2\Delta_\mathrm{m}^2}{\Omega_\mathrm{m}^2}}\right]. \tag{82}$$

From this we can calculate the mechanical amplitude as $|x| = \sqrt{x_1^2 + x_2^2}$ and get

$$|x|_\mathrm{on} = |x|_\mathrm{off}\left[\frac{\cos^2(\phi_\mathrm{p} + \varphi)}{\left(1 + \frac{V_{2\Omega}}{V_\mathrm{t}}\right)^2} + \frac{\sin^2(\phi_\mathrm{p} + \varphi)}{\left(1 - \frac{V_{2\Omega}}{V_\mathrm{t}}\right)^2}\right]^{1/2} \tag{83}$$

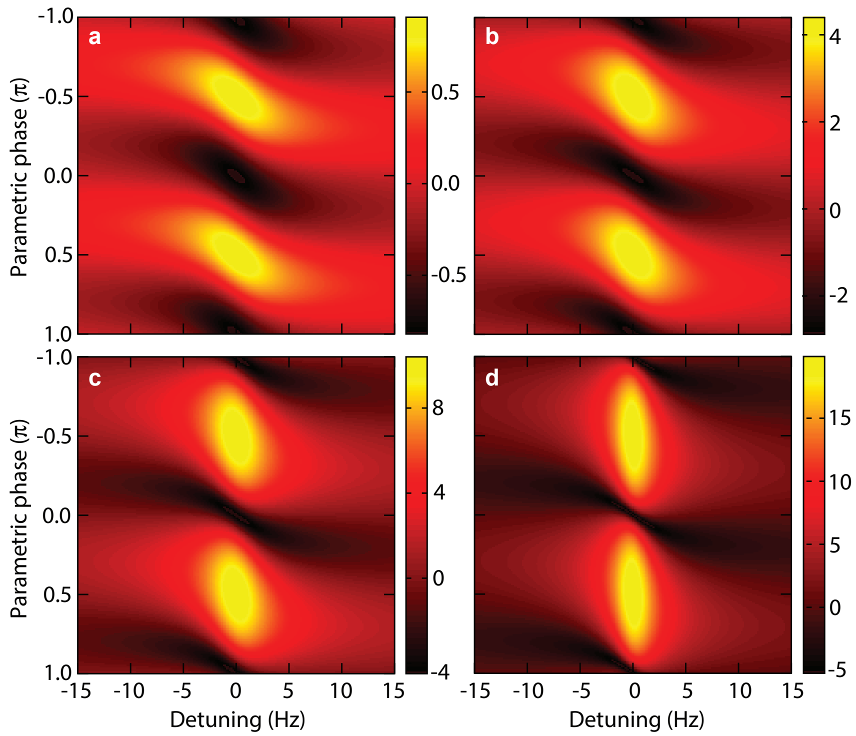

Supplementary Figure 8. **Calculated mechanical parametric gain.** Plots show the mechanical parametric gain vs parametric phase ϕ_p and vs detuning from the mechanical resonance frequency Ω_m for four different parametric modulation amplitudes. **a** $V_{2\Omega}/V_\mathrm{t} = 0.1$, **b** $V_{2\Omega}/V_\mathrm{t} = 0.4$, **c** $V_{2\Omega}/V_\mathrm{t} = 0.7$ and **d** $V_{2\Omega}/V_\mathrm{t} = 0.9$. The gain shows a π-periodicity and maxima/minima values follow an arctangent function with detuning. The calculation parameters were chosen close to the experimental device with $\Omega_\mathrm{m} = 2\pi \cdot 1.4315\,\mathrm{MHz}$ and $Q_\mathrm{m} = 195000$, the numbers at the color bars represent gain values in dB.

where the amplitude without parametric drive is given by the usual expression

$$|x|_\mathrm{off} = F_0 \frac{Q_\mathrm{m}}{k_0} \frac{1}{\sqrt{1+\frac{4Q_\mathrm{m}^2\Delta_\mathrm{m}^2}{\Omega_\mathrm{m}^2}}} \tag{84}$$

describing the square root of a Lorentzian line around the resonance frequency.

Equation (83) is a generalization of the expression given in Ref. [1] for non-zero detunings from the resonance frequency. The threshold voltage for parametric instability V_t' in Eq. (83) describes the square root of an inverted Lorentzian

$$V_\mathrm{t} = V_{\mathrm{t}0}\sqrt{1+\frac{4Q_\mathrm{m}^2\Delta_\mathrm{m}^2}{\Omega_\mathrm{m}^2}}, \tag{85}$$

increasing with detuning from the resonant value

$$V_{\mathrm{t}0} = \frac{2k_0}{Q_\mathrm{m} V_\mathrm{dc} \frac{\partial^2 C_\mathrm{nw}}{\partial x^2}}, \tag{86}$$

and the additional phase φ appearing originates from the usual phase shift between the driving force and the mechanical response in absence of a parametric modulation. It is given by

$$\varphi = -\frac{1}{2}\arctan\left(\frac{2Q_\mathrm{m}\Delta_\mathrm{m}}{\Omega_\mathrm{m}}\right). \tag{87}$$

Finally, we can give the expression for the mechanical gain as

$$G = \frac{|x|_{\mathrm{on}}}{|x|_{\mathrm{off}}} = \left[\frac{\cos^2(\phi_{\mathrm{p}} + \varphi)}{\left(1 + \frac{V_{2\Omega}}{V_{\mathrm{t}}}\right)^2} + \frac{\sin^2(\phi_{\mathrm{p}} + \varphi)}{\left(1 - \frac{V_{2\Omega}}{V_{\mathrm{t}}}\right)^2} \right]^{1/2} \tag{88}$$

with the maximum and minimum values

$$G_{\mathrm{max}} = \frac{1}{1 - \frac{V_{2\Omega}}{V_{\mathrm{t}}}}, \qquad G_{\mathrm{min}} = \frac{1}{1 + \frac{V_{2\Omega}}{V_{\mathrm{t}}}} \tag{89}$$

for

$$\phi_p^{\mathrm{max}} = \frac{\pi}{2} - \varphi, \qquad \phi_p^{\mathrm{min}} = -\varphi, \tag{90}$$

respectively.

Supplementary Fig. 8 shows the parametric mechanical gain as a function of parametric phase ϕ_{p} and detuning from the mechanical resonance frequency $\Delta_{\mathrm{m}} = \Omega - \Omega_{\mathrm{m}}$, calculated using Eq. (88). The different panels show the amplitude gain for four different ratios of parametric drive voltage to threshold voltage $V_{2\Omega}/V_{\mathrm{t}}$. As expected from the equations, we find a π-periodicity of the gain and the maximum and minimum values follow an arctangent as a function of detuning from the resonance frequency. We also see that with increasing parametric drive voltage and gain, respectively, the gradient of the gain with detuning also increases, demonstrating that frequency instabilities of the mechanical oscillator will lead to strong fluctuations of the gain as well in the high gain regime.

SUPPLEMENTARY NOTE 7: ADDITIONAL ANALYSES ON THERMOMECHANICAL NOISE SQUEEZING

Power spectral density analysis of the individual quadratures

To obtain the power spectral densities of the individual quadratures, we calculate and smooth $S_X = |X(\Omega)|^2$ and $S_Y = |Y(\Omega)|^2$ analogously and normalize them using the background noise of the corresponding quadrature. The result is plotted in Supplementary Fig. 9**a** and demonstrates how the noise power of the amplified Y-quadrature is increased and the corresponding power of the de-amplified X-quadrature is reduced with respect to the case without parametric driving. We fit the curve for $r = 0$ with a Lorentzian to extract the amplitude and linewidth $\Gamma_{\mathrm{th}} = 2\pi \cdot 9.2\,\mathrm{Hz}$ of the PSD without parametric driving, shown as black line on top of the Y_{off} data in Supplementary Fig. 9**a**.

According to Ref. [2], the individual quadrature power spectral densities are given by

$$S_X(r) = \frac{2k_{\mathrm{B}}T_X(r)}{k} \frac{\Gamma_{\mathrm{m}}}{4\Delta_{\mathrm{m}}^2 + \Gamma_{\mathrm{m}}^2(1+r)^2} \tag{91}$$

$$S_Y(r) = \frac{2k_{\mathrm{B}}T_Y(r)}{k} \frac{\Gamma_{\mathrm{m}}}{4\Delta_{\mathrm{m}}^2 + \Gamma_{\mathrm{m}}^2(1-r)^2} \tag{92}$$

with $r = V_{2\Omega}/V_{\mathrm{t}}$ and Δ_{m} the detuning from the mechanical resonance frequency. In these equations, we take into account possible quadrature-dependent added noise, which leads to an increase of the effective quadrature temperature $T_X(r)$ and $T_Y(r)$ by parametric driving, respectively. We can use these equations to model the spectral densities with parametric driving shown in Supplementary Fig. 9**a** and get good agreement for a wide range of parameters for $r = 0.65...0.75$, depending on the corresponding effective temperatures $T_X(r)$ and $T_Y(r)$. We note that for the possible value range of r, we find $T_X(r)/T_{\mathrm{m}} > 1$ and $T_Y(r)/T_{\mathrm{m}} > 1$ where T_{m} is the quadrature temperature without parametric driving. This means that the parametric driving adds excess noise to both quadratures, but from the PSD analysis alone, we cannot reliably determine the individual quadrature contributions.

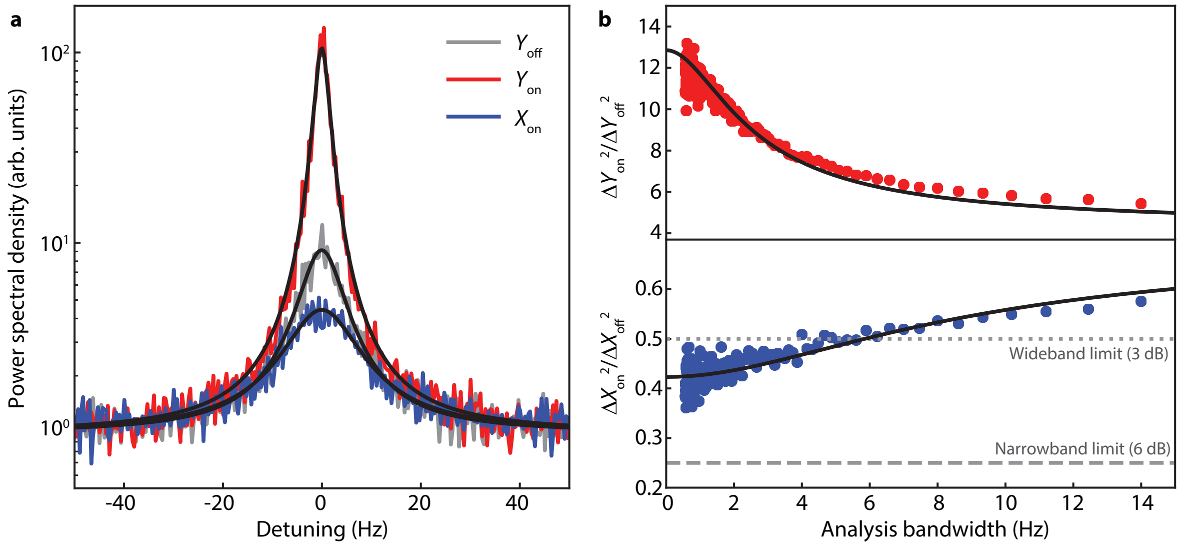

Supplementary Figure 9. **Quadrature-resolved noise analysis with parametric driving. a** Power spectral density of the individual quadratures calculated via $|X_{\rm on}(\Omega)|^2$ and $|Y_{\rm on}(\Omega)|^2$. For comparison, we also plot $|Y_{\rm off}(\Omega)|^2$, the power spectral density of the Y-quadrature without parametric driving. Colored lines are data, black lines are theoretical curves. **b** shows the variance ratio $\Delta X_{\rm on}^2/\Delta X_{\rm off}^2$ and $\Delta Y_{\rm on}^2/\Delta Y_{\rm off}^2$ for both quadratures vs the effective analysis bandwidth. Disks are data, black lines are theoretical curves. In the bottom panel, two horizontal dashed lines indicated the variance squeezing limits for $r \to 1$ in the narrowband case (6 dB) and in the wideband case (3 dB). The theoretical lines for the amplified and de-amplified quadrature and variances in **a** and **b** correspond to a single parameter set of $r = 0.67$, , $T_X/T_{\rm m} = 1.18$, $T_Y/T_{\rm m} = 1.4$, for details see text.

Histogram and variance analysis

The variances of the X- and Y-quadratures $\Delta X_{\rm on}^2$ and $\Delta Y_{\rm on}^2$ with parametric driving for an ideal amplification are given by [2]

$$\Delta X_{\rm on}^2 = \frac{1}{2\pi}\int_{-\infty}^{+\infty} S_X(\Delta_{\rm m})d\Delta_{\rm m} = \frac{\Delta X_{\rm off}^2}{1+r} \tag{93}$$

$$\Delta Y_{\rm on}^2 = \frac{1}{2\pi}\int_{-\infty}^{+\infty} S_Y(\Delta_{\rm m})d\Delta_{\rm m} = \frac{\Delta Y_{\rm off}^2}{1-r}. \tag{94}$$

If the bandwidth BW of the measurement or the calculation is finite, the integration span does not extend from $-\infty$ to $+\infty$, but from $-\mathrm{BW}/2$ to $+\mathrm{BW}/2$, which considerably impacts the squeezing factor $s = \Delta X_{\rm on}^2/\Delta X_{\rm off}^2$. It is straightforward to numerically integrate Eqs. (94) for a limited bandwidth. To compare the result with the experimental data for varying bandwidth, we apply bin averaging to $X(t)$ and $Y(t)$ using n bins from $n_{\rm min} = 8$ to $n_{\rm max} = 200$. The effective analysis bandwidth is given by $112.5\,\mathrm{Hz}/n$. We do not use numbers smaller than 8 here, as above a bandwidth of around 10 Hz the lock-in detection bandwidth deteriorates the results, and not larger than 200, because this would reduce our sample number to values for which the variances come with a large error. For each of the bin averaged time series, we numerically calculate the variances σ_X and σ_Y for parametric driving, no parametric driving and for the independently measured detection chain noise. The amplifier background noise is substracted from each dataset and we get $\Delta X^2 = \sigma_X^2 - \sigma_{{\rm amp},X}^2$ and $\Delta Y^2 = \sigma_Y^2 - \sigma_{{\rm amp},Y}^2$. From here, we calculate the variance ratios

$$\frac{\Delta X_{\rm on}^2}{\Delta X_{\rm off}^2}, \quad \frac{\Delta Y_{\rm on}^2}{\Delta Y_{\rm off}^2} \tag{95}$$

which are plotted as points in Supplementary Fig. 9**b** vs the effective analysis bandwidth. As black lines, the result of the numerical integration of Eqs. (94) is plotted for $r = 0.67$, $T_X/T_{\rm m} = 1.18$, $T_Y/T_{\rm m} = 1.4$. This set of parameters gives good agreement for both, the analysis of the power spectral densities and the bandwidth dependence of the quadratures. Therefore, the effective temperature of the de-amplified quadrature is increased by about 18% and of the amplified quadrature by about 40%. A possible source for the observed heating is resonance frequency fluctuations

of the nanobeam, which we indeed observed during our experiments. We estimate the possible uncertainty to be still around 10%, i.e., $r = 0.67 \pm 0.07$, as the two analysis methods lead to the best individual agreement between theory and experiment for $r_{\mathrm{PSD}} \approx 0.71$ and $r_{\mathrm{var}} \approx 0.61$. Deviations between theory and experiment are most probably due to the aforementioned mechanical frequency fluctuations and the lock-in amplifier filter function, which is not taken into account into the theoretical treatment.

SUPPLEMENTARY NOTE 8: THEORY OF PARAMETRIC MICROWAVE AMPLIFICATION

Equations of motion and general solution

We include the parametric driving equivalently to the case of mechanical parametric amplification into the optomechanical equations of motion and get

$$\delta\ddot{x} = -\left[\Omega_{\mathrm{m}}^2 + \Omega_{\mathrm{p}}^2 \cos\left(2\Omega t + \phi_{\mathrm{t}}\right)\right]\delta x - \Gamma_{\mathrm{m}}\delta\dot{x} + \frac{\hbar G_0 \bar{\alpha}}{m}\left(\delta\alpha + \delta\alpha^*\right) \tag{96}$$

$$\delta\dot{\alpha} = \left[i\bar{\Delta} - \frac{\kappa}{2}\right]\delta\alpha + iG_0\bar{\alpha}\delta x + \sqrt{\frac{\kappa_{\mathrm{e}}}{2}}S_{\mathrm{p}}. \tag{97}$$

where $\Omega_{\mathrm{p}}^2 = k_{\mathrm{p}}/m$ and ϕ_{t} considers an additional possible phase shift. We solve these equations again with the Ansatz

$$\delta\alpha = a_- e^{-i\Omega t} + a_+ e^{+i\Omega t} \tag{98}$$

$$\delta\alpha^* = a_-^* e^{+i\Omega t} + a_+^* e^{-i\Omega t} \tag{99}$$

$$\delta x = x_1 e^{-i\Omega t} + x_1^* e^{+i\Omega t} \tag{100}$$

and the identity

$$\cos\left(2\Omega t + \phi_{\mathrm{t}}\right) = \frac{1}{2}\left[e^{+i2\Omega t}e^{+i\phi_{\mathrm{t}}} + e^{-i2\Omega t}e^{-i\phi_{\mathrm{t}}}\right]. \tag{101}$$

Using rotating wave approximation and algebra yields the solution

$$x_1 = \hbar G_0 \bar{\alpha}\chi_{\mathrm{m}}^{\mathrm{eff}}\left[\frac{\chi_{\mathrm{c}} - \left(\chi_{\mathrm{m}}^{\mathrm{eff}}\right)^* \chi_{\mathrm{c}}^* \frac{m\Omega_{\mathrm{p}}^2}{2} e^{-i\phi_{\mathrm{t}}}}{1 - |\chi_{\mathrm{m}}^{\mathrm{eff}}|^2 \frac{m^2\Omega_{\mathrm{p}}^4}{4}}\right]\sqrt{\frac{\kappa_{\mathrm{e}}}{2}}S_0 \tag{102}$$

$$a_- = \chi_{\mathrm{c}}\left[1 + i2m\Omega_{\mathrm{m}}g^2\chi_{\mathrm{m}}^{\mathrm{eff}}\frac{\chi_{\mathrm{c}} - \left(\chi_{\mathrm{m}}^{\mathrm{eff}}\right)^* \chi_{\mathrm{c}}^* \frac{m\Omega_{\mathrm{p}}^2}{2} e^{-i\phi_{\mathrm{t}}}}{1 - |\chi_{\mathrm{m}}^{\mathrm{eff}}|^2 \frac{m^2\Omega_{\mathrm{p}}^4}{4}}\right]\sqrt{\frac{\kappa_{\mathrm{e}}}{2}}S_0. \tag{103}$$

This can be significantly simplified for drives on one of the sidebands, i.e., for $\omega_{\mathrm{d}} = \omega_{\mathrm{c}} \pm \Omega_{\mathrm{m}}$ and a probe very close to the cavity resonance $\Omega_{\mathrm{p}} \sim \omega_{\mathrm{c}}$.

Drive on the red sideband

When the cavity drive is set to the red sideband $\omega_{\mathrm{d}} = \omega_{\mathrm{c}} - \Omega_{\mathrm{m}}$ and the probe tone is sweeping only very close to the cavity resonance frequency $\Omega_{\mathrm{p}} = \omega_{\mathrm{c}} + \Delta_{\mathrm{m}}$ with $\Delta_{\mathrm{m}} \ll \kappa$, we can significantly simplify the equations. The cavity susceptibility becomes $\chi_{\mathrm{c}} = \chi_{\mathrm{c}}^* = 2/\kappa$ and the effective mechanical susceptibility becomes

$$\chi_{\mathrm{m}}^{\mathrm{eff}} = -\frac{1}{m\Omega_{\mathrm{m}}}\frac{1}{2\Delta_{\mathrm{m}} + i\Gamma_{\mathrm{eff}}}. \tag{104}$$

After introducing $\chi_{\mathrm{m}}^{\mathrm{eff}} = |\chi_{\mathrm{m}}^{\mathrm{eff}}|e^{i\varphi_{\mathrm{m}}}$ and the parameter

$$B = \frac{\Omega_{\mathrm{p}}^2}{2\Omega_{\mathrm{m}}}\frac{1}{\sqrt{\Gamma_{\mathrm{eff}}^2 + 4\Delta_{\mathrm{m}}^2}} = |\chi_{\mathrm{m}}^{\mathrm{eff}}|\frac{m\Omega_{\mathrm{p}}^2}{2} \tag{105}$$

we can rewrite the intracavity amplitude as

$$a_- = \frac{2}{\kappa}\left[1 - i\frac{\mathcal{C}\Gamma_{\mathrm{m}}}{2\Delta_{\mathrm{m}} + i\Gamma_{\mathrm{eff}}}\frac{1 - Be^{-i(\phi_{\mathrm{t}} + \varphi_{\mathrm{m}})}}{1 - B^2}\right]\sqrt{\frac{\kappa_{\mathrm{e}}}{2}}S_0 \tag{106}$$

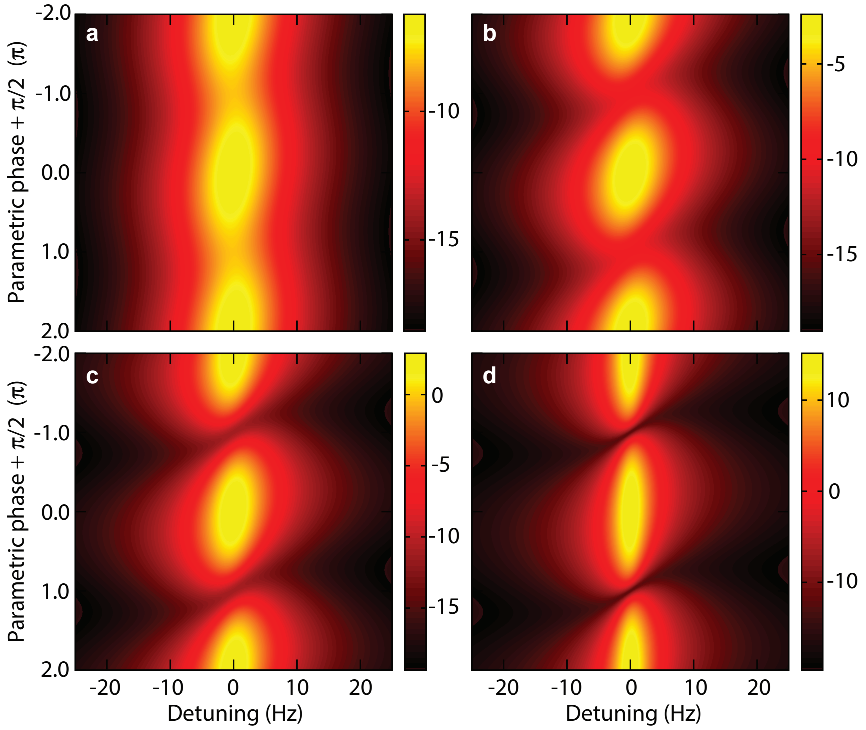

Supplementary Figure 10. **Calculated OMIT microwave transmission with mechanical parametric driving.** Plots show the microwave transmission signal S_{21} in dB vs parametric offset phase $\phi_t - \pi/2$ and vs detuning from the mechanical resonance frequency Ω_m for four different parametric modulation amplitudes. **a** $V_{2\Omega}/V_t = 0.12$, **b** $V_{2\Omega}/V_t = 0.46$, **c** $V_{2\Omega}/V_t = 0.72$ and **d** $V_{2\Omega}/V_t = 0.93$. The transmission shows a 2π-periodicity in parametric modulation phase. The calculation parameters were chosen close to the experimental device with $\kappa_i = 2\pi \cdot 370\,\text{kHz}$, $\kappa_e = 2\pi \cdot 5.6\,\text{MHz}$, $\Omega_m = 2\pi \cdot 1.4315\,\text{MHz}$, $\mathcal{C} = 0.5$ and $Q_m = 146000$, the numbers at the color bars represent the transmission parameter S_{21} in dB. In **c** and **d** a microwave signal going to the device experiences a net amplification.

and the transmission as

$$S_{21} = \frac{\kappa_i}{\kappa} + i\frac{\kappa_e}{\kappa}\frac{\mathcal{C}\Gamma_m}{2\Delta_m + i\Gamma_{\text{eff}}}\frac{1 - Be^{-i(\phi_t+\varphi_m)}}{1 - B^2}. \tag{107}$$

For the minimum and maximum transmitted power exactly on resonance we get

$$|S_{21}|^2 = \frac{\kappa_i^2}{\kappa^2} + \frac{\mathcal{C}_p\Gamma_m}{\Gamma_{\text{eff}}^2}\left[2\frac{\kappa_i\kappa_e}{\kappa^2}\Gamma_{\text{eff}} + \frac{\kappa_e^2}{\kappa^2}\mathcal{C}_p\Gamma_m\right], \tag{108}$$

which is the same equation as without parametric drive, but with a parametrically enhanced/reduced cooperativity

$$\mathcal{C}_p = \frac{\mathcal{C}}{1 \pm B_0} \tag{109}$$

with $B_0 = \Omega_p^2/2\Omega_m\Gamma_m$. The net microwave power gain in this regime is given by $G_{mw} = |S_{21}|^2_{max} - 1$. It is interesting to notice that the parameter B we introduced here, corresponds exactly to the voltage ratio $V_{2\Omega}/V_t'(\Gamma_{\text{eff}})$, cf. Supplementary Note 6, but with a threshold voltage determined by the effective mechanical linewidth. This means that the parametric instability regime onset is modified by the optomechanical interaction.

In Supplementary Fig. 10 we plot the result of Eq. (107) for four different parametric modulation strengths. The calculation parameters are chosen to be close to the device parameters, i.e., $\kappa_i = 2\pi \cdot 370\,\text{kHz}$, $\kappa_e = 2\pi \cdot 5.6\,\text{MHz}$, $\Omega_m = 2\pi \cdot 1.4315\,\text{MHz}$, $\mathcal{C} = 0.5$ and $Q_m = 146000$, where the latter is adjusted to the value we expect for the corresponding sideband drive power, cf. Supplementary Fig. 7**a**. For small modulation $V_{2\Omega}/V_t = 0.12$ as shown in **a**, the OMIT signal is only slightly distorted from the signal without parametric modulation, but the phase sensitivity of the amplifier becomes already apparent. For larger parametric modulations, the maximum gain increases until it

reaches $\sim 15\,\mathrm{dB}$ for $V_{2\Omega}/V_{\mathrm{t}} = 0.93$ as shown in **d**. Linecuts for the phase-dependence at zero detuning and the lines of maximum gain for each power are shown in Supplementary Fig. 11**a** and **b**.

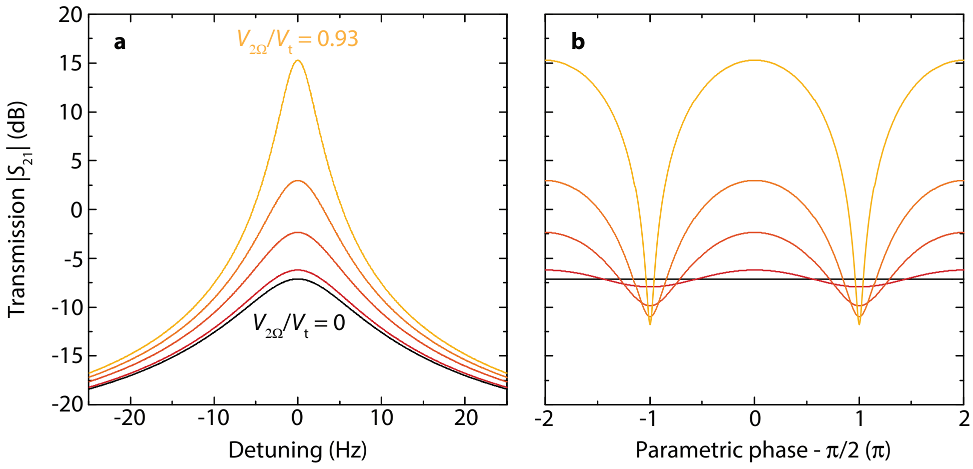

Supplementary Figure 11. **Maximum gain and phase sensitivity of the amplification.** In **a** we show the maximum gain extracted from the calculated data in Supplementary Fig. 10, the lowest curve shows the OMIT signal without parametric drive. Note that for each detuning, the absolute phase to reach the shown maximum is different. In **b** we plot the phase dependence of the transmitted signal for zero detuning, which corresponds to vertical linecuts through the panels of Supplementary Fig. 10.

Note that the phase periodicity here is 2π, which is a consequence of including the parametric phase into the parametric drive in the theoretical treatment here instead of the driving force. In addition, we have to consider a formal phase lag of $\pi/2$ induced by the phase of the cavity response function, which on resonance vanishes, while the phase of a directly driven mechanical oscillator on resonance is $-\pi/2$.

Drive on the blue sideband

On the blue sideband, we get $\chi_{\mathrm{m}}^{\mathrm{eff}} = -|\chi_{\mathrm{m}}^{\mathrm{eff}}|e^{i\varphi_{\mathrm{m}}}$ and thus

$$a_{-} = \frac{2}{\kappa}\left[1 + i\frac{\mathcal{C}\Gamma_{\mathrm{m}}}{2\Delta_{\mathrm{m}} + i\Gamma'_{\mathrm{eff}}}\frac{1 + Be^{-i(\phi_{\mathrm{t}}+\varphi_{\mathrm{m}})}}{1 - B^2}\right]\sqrt{\frac{\kappa_{\mathrm{e}}}{2}}S_0 \tag{110}$$

for the intracavity field. The transmission parameter becomes

$$S_{21} = \frac{\kappa_{\mathrm{i}}}{\kappa} - i\frac{\kappa_{\mathrm{e}}}{\kappa}\frac{\mathcal{C}\Gamma_{\mathrm{m}}}{2\Delta_{\mathrm{m}} + i\Gamma'_{\mathrm{eff}}}\frac{1 + Be^{-i(\phi_{\mathrm{t}}+\varphi_{\mathrm{m}})}}{1 - B^2} \tag{111}$$

One example for the gain obtained when driving on the blue sideband is given in Supplementary Fig. 12**a**. The phase sensitivity and the total gain is comparable to the values obtained for a drive on the red sideband for a similar value of $V_{2\Omega}/V_{\mathrm{t}} = 0.93$ with an additional phase shift of π in the parametric phase dependence. In **b**, the maximum microwave transmission is shown as blue line and the bare transmission signal without parametric drive as black line for this parameter regime. For comparison, the equivalent data for the red sideband drive are shown as dashed lines.

We note that although the relative parametric pump strength is comparable for the red and the blue detuned drive here, the absolute numbers are different, due to the smaller effective mechanical linewidth in the blue-detuned case.

SUPPLEMENTARY NOTE 9: ADDITIONAL DATA IN THE NONLINEAR MECHANICAL REGIME

In the main paper Fig. 4, the net microwave gain is limited to around 7 dB, corresponding to an intracavity field gain of about $\sim 14\,\mathrm{dB}$. The device is also capable of generating larger gain of up to $12\,\mathrm{dB}$ net gain and about $19\,\mathrm{dB}$ intracavity gain, but due to the experimental parameters used for these measurements, the mechanical resonator enters the nonlinear regime for these high gain values. Due to the relatively large probe tone used for the measurement

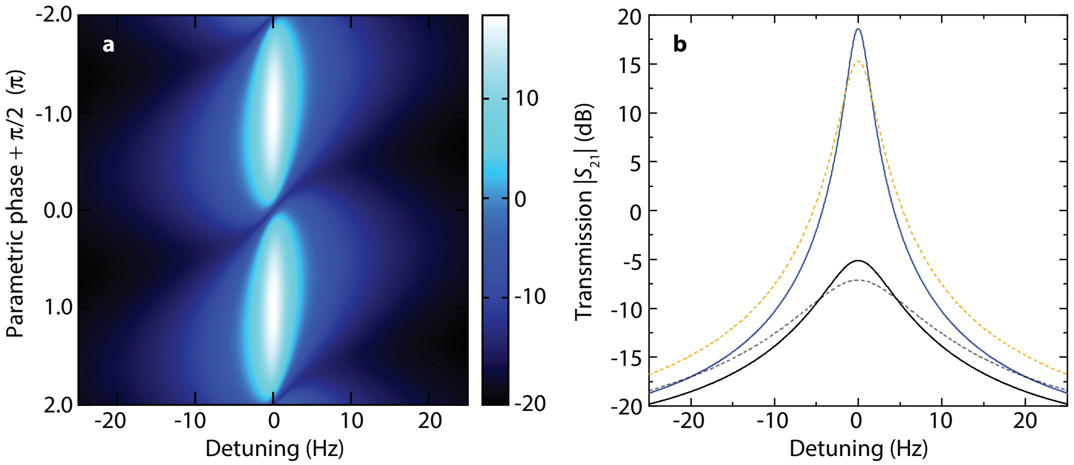

Supplementary Figure 12. **Phase-sensitive amplification with a drive on the blue sideband.** In **a** we show the cavity transmission $|S_{21}|$ in dB for a drive on the blue sideband and a parametric excitation amplitude $V_{2\Omega}/V_\mathrm{t} \approx 0.93$. All other parameters are as given in the caption of Supplementary Fig. 10. In **b** we show as solid lines the window of maximum transmission without parametric drive (black) and with parametric drive (blue) as extracted from **a**. The dashed lines show the equivalent curves for a red detuned drive.

(intracavity probe photons $\sim$ 7000), the mechanical resonator has a large starting amplitude and is entering the nonlinear regime for parametric drive voltages above $V_\mathrm{t}/V_{2\Omega} > 0.75$. The increased gain and the nonlinear mechanical resonance are shown in Supplementary Fig. 13**a**, showing a clear Duffing-like OMIT resonance curve, a maximum intracavity gain of $\sim 19\,\mathrm{dB}$, and a net gain of $\sim 12\,\mathrm{dB}$.

In panels **b-g**, the parametric phase dependence of the gain for different detunings is shown, the frequency points are indicated in **a**. For negative detunings, the phase dependence looks very similar to the linear regime, cf. **b** and **c**. For the detuning shown in **c**, something new happens. Depending on the parametric phase (i.e. on the expected linear gain), the transmission shows sharp transitions between the mechanical low- and high-amplitude branches, indicating the Duffing regime of the mechanical oscillator. The transmission is still 2π-periodic, but it is not symmetric with respect to phase inflections anymore. This trend is still somewhat visible in **d**, although at the corresponding detuning ($\Delta_\mathrm{m} \approx 0$, smaller the critical detuning) there is only one amplitude state available and so no sharp transitions occur. For increasing positive detuning, the phase-dependence approaches the behaviour of a linear oscillator again, cf. **f** and **g**.

SUPPLEMENTARY NOTE 10: APPROACH FOR AN OPTIMIZED DEVICE

There is plenty of space for optimizations regarding a possible future device, which can lead to near quantum-limited phase-sensitive microwave amplification with increased bandwidth and a net microwave gain of $\sim 20\,\mathrm{dB}$. Most important is to increase the cooperativity $\mathcal{C} = \frac{4n_\mathrm{c}g_0^2}{\kappa\Gamma_\mathrm{m}}$, which can be achieved by increasing the single-photon coupling rate g_0. In order to enable the resolved sideband limit for efficient cooling of the mechanical oscillator, it will also be helpful to reduce the cavity linewidth κ significantly. The current linewidth is predominantly given by the external linewidth, which is dominated by the non-mechanical coupling capacitance.

Hence, the most straightforward way to achieve smaller linewidth and at the same time larger g_0 is to reduce both, the coupling capacitance by a factor of ~ 5 and the resonator capacitance by about a factor of ~ 10, which should be easily possible using a lumped element resonator. This will at the same time reduce the external cavity linewidth to about $\kappa_\mathrm{e} = 2\pi \cdot 250\,\mathrm{kHz}$ and increase the coupling rate to $g_0 = 2\pi \cdot 10\,\mathrm{Hz}$, leading to a cooperativity of about ~ 500 for intracavity photon numbers comparable to the current experiment. For this, we assume an internal cavity linewidth of $\kappa_\mathrm{i} = 2\pi \cdot 250\,\mathrm{kHz}$, which also will put the device into the resolved sideband limit with $\Omega_\mathrm{m}/\kappa \sim 3$. If the internal linewidth can be further reduced to a realistically low value of about 50 kHz, both the cooperativity and the sideband-resolution factor will profit.

In the high-cooperativity regime $\mathcal{C} \gg 1$, the bandwidth will be significantly increased by means of optical damping $\Gamma_\mathrm{eff} = \Gamma_\mathrm{m}(1+\mathcal{C})$ and the OMIT window will show a transmission of $|S_{21}| \approx 1$ without parametric drive, which means that any intracavity field gain will translate to net signal gain. With this configuration, it seems feasible to achieve the near-quantum-limited phase-sensitive amplification regime with a net signal gain of $\sim 20\,\mathrm{dB}$ and a bandwidth of

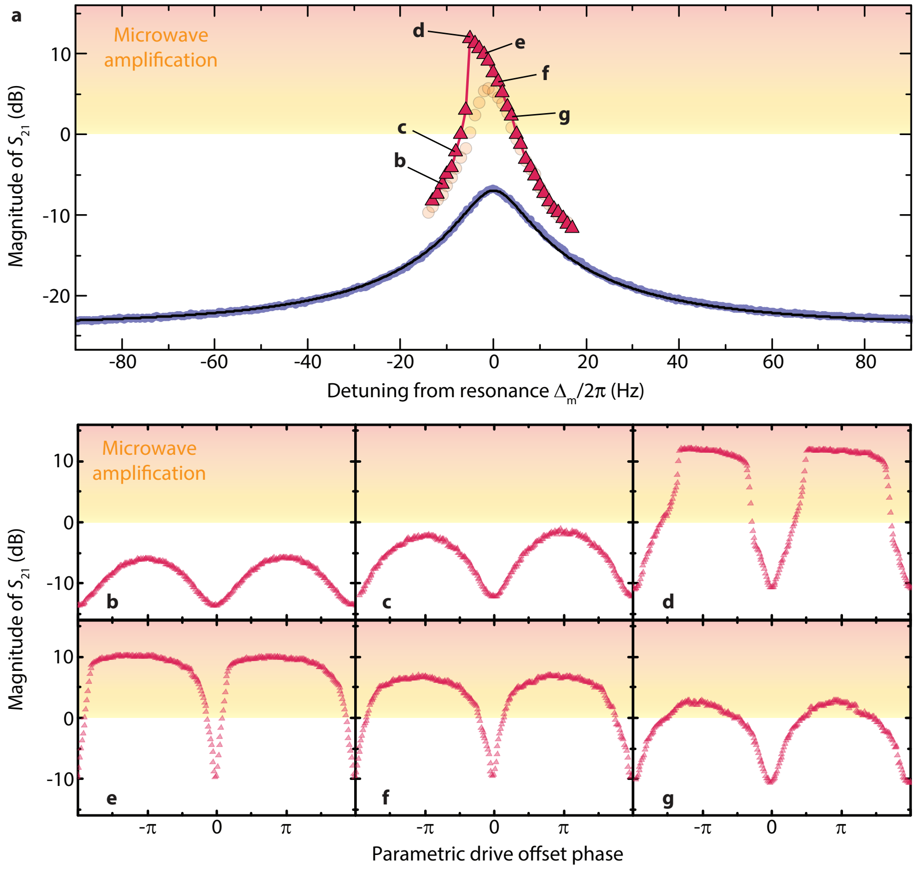

Supplementary Figure 13. **Phase-sensitive microwave amplification with the mechanical oscillator in the nonlinear regime. a** shows the cavity transmission $|S_{21}|$ at $\omega \approx \omega_c$ for a red-sideband drive tone with and without parametric drive. Blue dots and black Lorentzian fit are the OMIT signal without any parametric modulation, the red triangles are the maximum value at each detuning for $V_{2\Omega}/V_t = 0.925$ (connecting lines are guide to the eye) and the transparent orange circles are for $V_{2\Omega}/V_t = 0.74$ as also shown in main paper Fig. 4**c**. **b**-**g** show the phase-sensitive microwave gain for different detunings from the OMIT resonance, points are marked in **a**. The experimental settings in this experiment are equivalent to the ones for main paper Fig. 4, the only difference is $V_{2\Omega}/V_t = 0.925$

$\sim$ kHz.

Supplementary References

[1] Rugar, D. and Grütter, P., Mechanical Parametric Amplification and Thermomechanical Noise Squeezing, Phys. Rev. Lett. **67**, 699 (1991)

[2] Vinante, A., and Falferi, P., Feedback-Enhanced Parametric Squeezing of Mechanical Motion, Physical Review Letters **111**, 207203 (2013).
